# PCMRESP: A Method for Polarizable Force Field Parameter Development and Transferability of the Polarizable Gaussian Multipole Models Across Multiple Solvents

**DOI:** 10.1021/acs.jctc.4c00064

**Published:** 2024-03-19

**Authors:** Yong Duan, Taoyu Niu, Junmei Wang, Piotr Cieplak, Ray Luo

**Affiliations:** † UC Davis Genome Center and Department of Biomedical Engineering, University of California, Davis, One Shields Avenue, Davis, California 95616, United States; ‡ Department of Pharmaceutical Sciences and Computational Chemical Genomics Screening Center, School of Pharmacy, University of Pittsburgh, Pittsburgh, Pennsylvania 15261, United States; § SBP Medical Discovery Institute, 10901 North Torrey Pines Road, La Jolla, California 92037, United States; ∥ Departments of Molecular Biology and Biochemistry, Chemical and Biomolecular Engineering, Materials Science and Engineering, and Biomedical Engineering, University of California, Irvine. Irvine, California 92697, United States

## Abstract

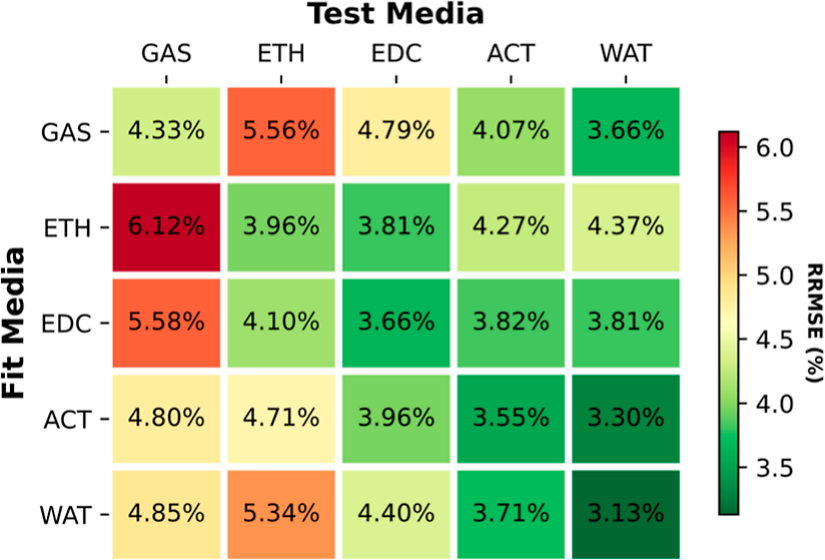

The transferability of force field parameters is a crucial aspect of high-quality force fields. Previous investigations have affirmed the transferability of electrostatic parameters derived from polarizable Gaussian multipole models (pGMs) when applied to water oligomer clusters, polypeptides across various conformations, and different sequences. In this study, we introduce PCMRESP, a novel method for electrostatic parametrization in solution, intended for the development of polarizable force fields. We utilized this method to assess the transferability of three models: a fixed charge model and two variants of pGM models. Our analysis involved testing these models on 377 small molecules and 100 tetra-peptides in five representative dielectric environments: gas, diethyl ether, dichloroethane, acetone, and water. Our findings reveal that the inclusion of atomic polarization significantly enhances transferability and the incorporation of permanent atomic dipoles, in the form of covalent bond dipoles, leads to further improvements. Moreover, our tests on dual-solvent strategies demonstrate consistent transferability for all three models, underscoring the robustness of the dual-solvent approach. In contrast, an evaluation of the traditional HF/6-31G* method indicates poor transferability for the pGM-ind and pGM-perm models, suggesting the limitations of this conventional approach.

## Introduction

In molecular mechanics force fields, the electrostatic components account for the long-range forces and are often approximated by the contributions up to the quadrupoles. The electrostatic components can potentially be one of the least transferable parts of a force field due to various approximations, for instance, those involving representation of the electrostatic potentials by limited terms up to quadrupoles. This is particularly true in the traditional point charge models, in which each atom is represented by a fixed point charge. The limited transferability hinders applications to systems that require changes in dielectric environments (e.g., involving large-scale conformational changes). To improve the transferability and enable accurate modeling of the electrostatic potentials across multiple solvation environments, polarizable force fields have been developed.

The induced dipole model is one of the extensively studied methods in which polarization is represented by the induced dipoles in response to the surrounding electrostatic environment. In this model, the induced dipoles are defined by eq 1
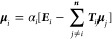
1where ***μ***_*i*_ represents the induced dipole of atom *i*, α_*i*_ is its polarizability, and *E*_*i*_ is the static electrostatic field acting on atom *i*. The dipole field tensor, ***T***_*ij*_, is given by eq 2.
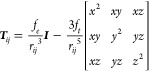
2here, *x*, *y,* and *z* are the Cartesian components of the distance vector between atoms *i* and *j*, *r*_*ij*_ is the distance, and ***I*** is the identity matrix. *f*_*e*_ and *f*_*t*_ denote the distance-dependent Thole^1,2^ damping functions that attenuate ***T***_*ij*_. These damping functions are crucial for preventing the “polarization catastrophe”, a problem encountered in classic Applequist point dipole models,^3,4^ where induced dipoles can reinforce each other and hinder convergence due to *f*_*e*_ and *f*_*t*_ both being equal to 1. With the distance-dependent damping functions, the induced dipole **μ**_*i*_ can remain finite when the atoms are in close contact. However, it is important to note that Thole models only attenuate induced dipole interactions while treating other electrostatic terms as interactions between point multipoles. This can lead to unphysically large electrostatic fields when the two atoms are in close contact. Furthermore, reconciling the short-range and long-range contributions in the Thole models, due to the presence of the nonlinear polarization energy term, , which requires full account of the electrostatic fields, including even the mostly static fields from bonded atoms, remains challenging without damping of other terms.

In a series of recent studies, we have introduced the polarizable Gaussian multipole model (pGM)^5−11^ based on the work of Elking et al.^12^ In this model, all multipoles are represented by Gaussian distribution functions,^12−14^ with the *n*th-order multipole defined by eq 3
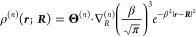
3

This formulation provides a uniform treatment of all multipoles and effectively eliminates the points that are the root causes of potential singularities while also coherently addressing the charge-penetration effect. The pGM model offers a comprehensive framework for accurately modeling electrostatic interactions in our research. In this framework, the damping functions are defined as follows
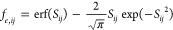
4
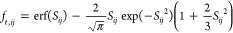
5
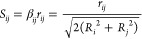
6

In these equations, *r*_*ij*_ is the distance between atoms *i* and *j*, and *R*_*i*_ and *R*_*j*_ are their respective pGM radii of the Gaussian functions.

## Theory

In recent developments, we introduced PyRESP,^15^ a python program designed for electrostatic parametrization in both polarizable and nonpolarizable force fields. Additionally, we introduced PyRESP_GEN,^11^ a companion tool for generating the input files for PyRESP. Building upon these foundations, our current work extends the capabilities of PyRESP to enable direct consideration of solvent polarizations in electrostatic parametrization using polarizable continuum model (PCM),^16^ which we call PCMRESP.

In PyRESP,^15^ we define the induced dipole vector ***μ*** = [***μ***_1_, ***μ***_2_, ...,***μ***_*n*_], consisting of individual atomic induced dipoles, ***μ***_1_, ***μ***_2_, ..., ***μ***_*n*_. The vector ***μ*** is related to the static electric field vector ***E*** given in eq 7
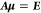
7where ***A*** is a 3*n* × 3*n* matrix whose diagonal entries are the inverse of atomic polarizabilities, and off-diagonal entries are the distance-dependent dipole tensors. ***E*** is a 3*n*-dimensional vector charactering the electric field generated by static charges *q* and permanent dipoles ***p***.

While considering the presence of PCM surface charges, the electrostatic field ***E***_*i*_ at a specific position *i* can be modified to account for the contributions from these surface charges *q*_*l*_, as given in eq 8. This modification is an essential aspect of our extended PyRESP, enabling the direct consideration of solvent polarizations in the electrostatic parametrization process.
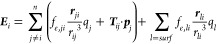
8here, *q*_*j*_ represents the charges of the *j*th atom, while *q*_*l*_ corresponds to the charges of the *l*th surface point. ***r***_*ji*_ and ***r***_*li*_ are the distance vectors, indicating the distances from the *j*th atom and *l*th surface point to the *i*th atom, respectively. The factors *f*_*e,ji*_ and *f*_*e,li*_ are defined in eq 4. Additionally, ***p***_*j*_ denotes the permanent dipoles and ***T***_*ij*_ represents the distance-dependent dipole field tensors as defined in eq 2. When the surface points are also represented as monopoles of Gaussian distributions, *f*_*e,li*_ can be calculated using the atomic radii and the radii associated with the surface charges. This inclusion of surface charges further enhances the accuracy and completeness of our model. The eq 9 succinctly represents eq 8 in the matrix form
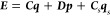
9

In this equation, ***E*** represents the electric field vector, ***C*** is a matrix of the charge field vectors associated with atomic charges ***q***, ***D*** is a matrix linked to permanent dipoles ***p*** and its elements are the dipole field tensors given in eq 2, and ***C***_*s*_ pertains to the matrix of the charge field vectors involving surface charges ***q***_*s*_.

It is worth noting that eq 9 introduces a notable difference from eq 30 in our earlier work,^15^ where surface charges were not considered. Here, ***C***_*s*_***q***_*s*_ signifies that the contribution of surface charge polarization which remains static in the fitting process and contributes to the induced dipoles. The electrostatic potential at position *j* outside the molecule can be calculated using eq 10, which involves the contributions of charges *q*_*i*_, induced dipoles ***μ***_*i*_, permanent dipoles ***p***_*i*_, and error function erf().
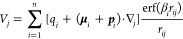
10here, β_*i*_ is similar to the one defined in eq 6 with *R*_*j*_ = 0. The abovementioned equation can be expressed in the matrix form as shown in eq 11 below
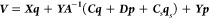
11
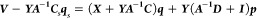
12where ***X*** is the matrix for the charge-electrostatic potential and ***Y*** is the matrix for the dipole-electrostatic potential.

Comparing eq 12 with eq 34 from our earlier work^15^ reveals an additional term ***YA***^−1^***C***_*s*_***q***_*s*_, which signifies the contribution of surface charge to the external electrostatic potentials while considering atomic polarizations. Following eq 36 from our previous work,^15^ we further express eq 12 in the form of eq 13 by introducing the matrix ***F*** to convert ***p***^*loc*^ from local covalent bond vectors (CBV)^6^ frame to ***p*** in global Cartesian frame.
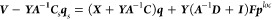
13

This transformation allows us to formulate the constrained least-squares fitting procedure, following the same steps outlined in our earlier work.^15^

## Methods

### Data Sets

In this study, we utilized two data sets: DES, comprising 377 small molecules, and TET-pep, consisting of 20 blocked tetra-peptides. Each tetra-peptide in the TET-pep data set was modeled as ACE-ALA-X-ALA-NME, where X represents a standard amino acid. The terminal ACE and NME denote the *N*-acetyl and *N*-methylamide terminal groups. Each tetra-peptide was modeled in five distinct conformations, representing the key mainchain conformations commonly found in proteins. These conformations include the antiparallel β-sheet (aβ), right-handed α-helix (αR), left-handed α-helix (αL), β-sheet (β), and polyproline type II (pII) conformations. Each molecule in the DES set is a single conformation.

For the TET-pep data set, the initial coordinates of the 100 tetra-peptide conformers were obtained from the work of Jiang et al.^17^ The coordinates were subsequently optimized at the MP2/6-311++G(d, p) level of theory, with fixed mainchain torsional angles (ϕ, ψ) set to (−140, 135°), (−57, −47°), (57, 47°), (−119, 113°), and (−79, 150°), corresponding to the five conformations.

Likewise, for the DES data set, the initial coordinates of the molecules were sourced from Shaw and the co-workers.^18,19^ These coordinates were also optimized at the MP2/6-311++G(d, p) level of theory for the sake of consistency. Both data sets were utilized for parametrization and subsequent testing in our study.

### Electrostatic Potentials

The electrostatic potentials were calculated in five solvents: gas (GAS), diethyl ether (ETH, ε = 4.24), dichloroethane (EDC, ε = 10.125), acetone (ACT, ε = 20.493), and water (WAT, ε = 78.3553). To account for solvent polarization effects, we employed the PCM.^16^ The surface was generated using Lebedev-Laikov grids, applying SMD-Coulomb atomic radii developed by Truhlar and co-workers.^20^ The grids were on the molecular surface with a density of approximately 5 points/Å^2^ and further smoothed using the York-Karplus algorithm.^21^ The surface polarization charges were represented as spherical Gaussians.

For the DES data set, the electrostatic potentials were calculated at the MP2/aug-cc-pVTZ level of theory. For the TET-pep data set, the electrostatic potentials were calculated at the ωB97-XD/aug-cc-pVTZ. Grid points for electrostatic potentials calculations were generated based on the method developed by Singh et al.^22,23^ These grid points were located at distances of 1.4, 1.6, 1.8, and 2.0 times the van der Waals radii, with a grid density of 6 points per Å^2^. All QM calculations were performed using the Gaussian 16 package.^24^ For reference, a sample input file for Gaussian calculations is included in the Supporting Information.

### Parameter Development and Test

The parameters of the DES data set, including atomic monopoles and permanent dipoles, were developed using a two-stage fitting procedure that involved iteratively fitting the electrostatic potentials, as extensively detailed in our prior publications.^11,15^ In the first stage, the initial monopoles were set to zero, and for the pGM-perm model, the initial permanent dipoles were also set to zero. During this stage, chemically equivalent atoms, except those in the –CH_2_– and –CH_3_ groups, were constrained to have identical parameters. In the second stage, only the –CH_2_– and –CH_3_ groups underwent fitting with appropriate chemical equivalencing applied. Other parameters, including monopoles and permanent dipoles, retained values obtained from the first stage of fitting. For the TET-pep data set, parameters were developed for each peptide by combining all five conformations in a single-stage procedure. In this process, chemical equivalence was enforced for all atoms except the methyl groups of the terminal residues. In PCM fitting, the surface charges, coordinates, and weighting factors were taken directly from Gaussian outputs. The chemical equivalencing in the fitting process is expected to lead to some degree of deterioration in the fitting quality because of the reduced number of degrees of freedom. However, because many of these groups can rotate freely, the chemical equivalencing effectively accounts for the averaging effects.

The primary objective of our transferability was to assess the extent to which electrostatic parameters obtained in one medium could be applied to other media. We selected the gas phase, diethyl ether, acetone, dichloroethane, and water as the test media, encompassing a range of dielectric constants from 1.0 to 78.36. All of the solution media were implicitly described using PCM as implemented in Gaussian 16 software.

We developed parameters for both single and dual solvents. In the case of dual solvents, the electrostatic potentials and surface charges from quantum mechanical calculations in two solvents were combined in each parametrization calculation. In this case, for each fitted molecule, two electrostatic potential files corresponding to two solvent media and standard inputs are combined in the same way as is done for the standard RESP multiconformational and multimolecular fitting process. Both single and dual solvent parameters were tested on the five single solvent electrostatic potentials. For the single solvent parameters, we applied the parameters to calculate electrostatic potentials in the media different from the one used in parametrization.

As a measure of errors, we calculated the root-mean-square error (RMSE) and relative RMSE (RRMSE) between calculated and quantum-mechanics-derived electrostatic potentials. These RMSEs and RRMSEs as transfer RMSEs and transfer RRMSEs, respectively, are measurements of transferability to distinguish them from those calculated during fitting. For dual solvent parameters, we conducted tests on all five individual media.

## Results and Discussion

### Fitting Quality Assessment

In our previous work, we investigated the quality and transferability of pGM models across various oligomeric states, conformations, and sequences, using water clusters and polypeptides as the model systems.^9^ In this study, we focus on assessing the transferability of electrostatic parameters among different solvents, specifically gas phase, diethyl ether, dichloroethane, acetone, and water. These solvents cover a wide range of dielectric constant values. All solvent media were modeled using PCM as implemented in Gaussian 16 software, and the electrostatic potentials were derived at either the MP2/aug-cc-pVTZ level (for the DES data set) or ωB97-XD/aug-cc-pVTZ level (for the TET-pep data set) of theory. To minimize the discrepancy between the QM and MM continuum solvent models, the surface point coordinates and charges were taken directly from the Gaussian output.

**Figure 1:**
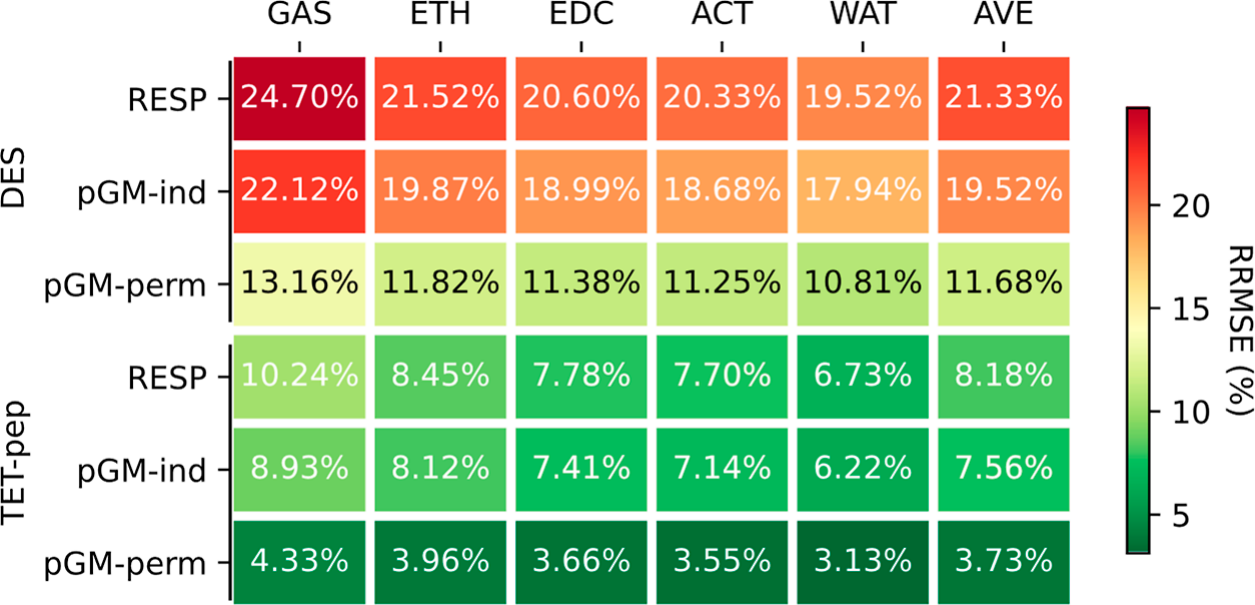
Average RRMSEs of the fitting.

The heat maps in Figures 1 and S1 in the Supporting Information depict the average relative root-mean-square errors (ARRMSE) and the average root-mean-square errors (ARMSE), respectively, for fitting three different models: RESP, pGM-ind, and pGM-perm models across five different solvent media. The RESP model, a fixed charge model representing electrostatic potentials using point monopoles, is the simplest and most widely used among the three. The pGM-ind represents the electrostatic potentials by a combination of fixed charge monopoles and induced dipoles, both in the form of Gaussian distributions, as described in eq 3. In the pGM-perm model, in addition to Gaussian monopoles and induced dipoles, permanent dipoles with Gaussian distributions are also employed.

Consistent with our earlier results, inclusion of induced dipoles enhances the fitting quality from the fixed charge models, regardless of the solvent environment, despite the fact that both models share an identical number of fitting parameters. However, the improvements were rather small for small molecule DES data set with single conformation. When averaged across all five solvent media, the ARRMSE and ARMSE for the point charge RESP fitting were 21.3% and 0.0026 au, respectively, for the DES data set. With Gaussian monopoles and Gaussian-induced dipoles, the pGM-ind model slightly reduced the relative and root-mean-square fitting errors to 19.5% and 0.0024 au, respectively. Notably, the pGM-perm model, a model with monopoles, induced, and permanent dipoles, all in Gaussian distributions, exhibited a significant improvement, reducing the average fitting errors to 11.7% and 0.0015 au, respectively, which are approximately half of those of RESP fitting. Similar observations were made for the tetra-peptides where the average RRMSE and RMSE for the point charge RESP fitting were 8.2% and 0.0027 au, respectively. These values improved to 7.6% and 0.0024 au, respectively, for pGM-ind and further improved to 3.7% and 0.0012 au, respectively, for pGM-perm. Therefore, our conclusion is that the inclusion of induced dipoles leads to slight improvement in fitting quality, and when permanent dipoles are added, the pGM-perm model outperforms both RESP and pGM-ind.

An interesting observation was that the average fitting RRMSEs consistently decreased with increasing solvent polarity. This trend held true for all three models and for both data sets. For RESP point charge fit, gas phase RRMSE was 24.7%, which decreased to 21.5, 20.6, 20.3, and 19.5% for ETH, EDC, ACT, and WAT, respectively, in the DES data set. When tested on the TET-pep data set, they were 10.2, 8.5, 7.8, 7.7, and 6.7%, respectively. For pGM-ind, they were 22.1, 19.9, 19.0, 18.7, and 17.9%, respectively, for the DES set and 8.9, 8.1, 7.4, 7.1, and 6.2%, respectively, for the TET-pep set. For pGM-perm, they were 13.2, 11.8, 11.4, 11.3, and 10.8%, respectively, for the DES set and 4.3, 4.0, 3.7, 3.6, and 3.1%, respectively, for the TET-pep set. This suggests that solvent polarization has an effect that makes the electrostatic potentials more consistent with those from the three models.

### Transferability in Single Solvent Models

In our previous work, we demonstrated that the pGM-perm model significantly enhances transferability across water clusters, poly-Ala and poly-Gly peptides, and heterosequence peptides and across multiple conformations. In this study, we extend our investigation to assess the transferability of the three models across various solvent media.

**Figure 2:**
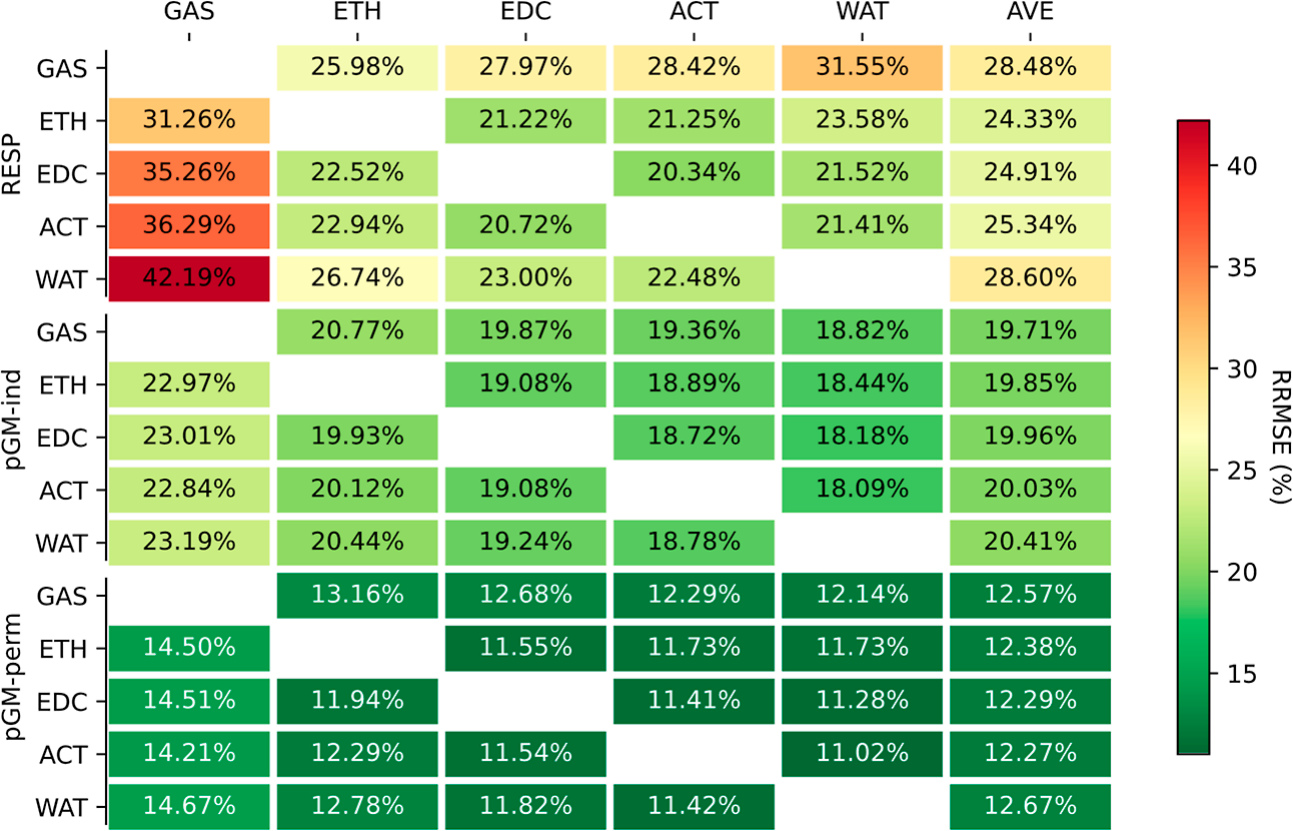
Transferability of RESP, pGM-ind, and pGM-perm models, as measured by the average RRMSE using the DES set. Each column is a tested solvent medium, and each row is a medium in which the parameters were developed.

**Figure 3:**
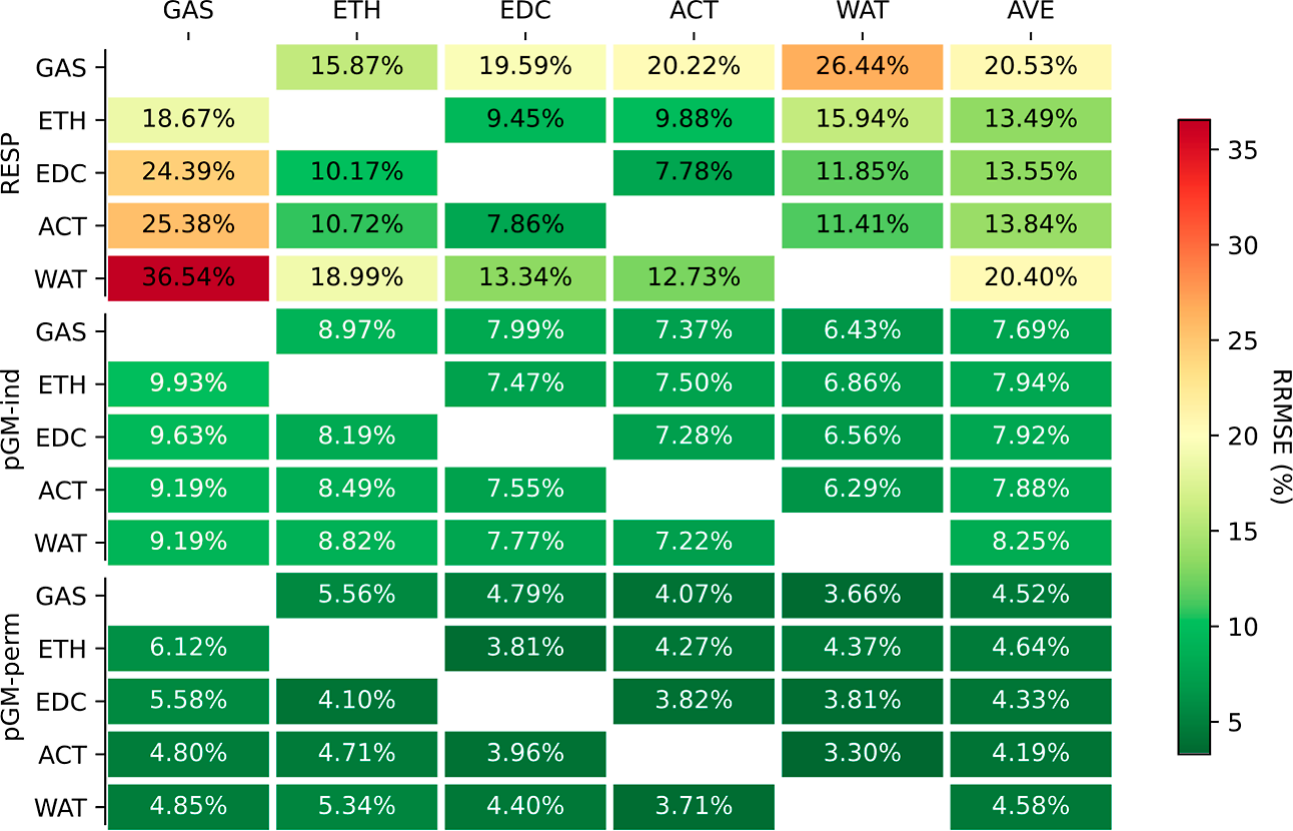
Transferability of RESP, pGM-ind, and pGM-perm models, as measured by the average transfer RRMSE using the TET-pep data set. Each column is the tested medium, and each row is the medium in which the parameters were developed.

Figures 2 and 3 present the ARRMSEs between the calculated ESPs using the fitted parameters and those derived from the quantum mechanical calculations in media different from those used for parameter development. The corresponding ARMSEs can be found in Figures S2 and S3 in the Supporting Information. Interestingly, although the pGM-ind model exhibited only a marginal improvement in fitting quality compared to RESP, it demonstrated consistent, albeit modest, enhancements in transferability across all tested media. On average, RESP parameters resulted in errors ranging from 24.3 to 28.6% when applied to calculate the ESPs for the DES data set in four different media not used in the fitting process. In contrast, the average relative errors of the pGM-ind parameters ranged from 19.7 to 20.4%. It is apparent that induced dipoles contribute to transferability, even though their role in reducing fitting errors were relatively small.

The most impressive transferability was achieved with the pGM-perm model. With permanent atomic dipoles aligned along bond directions, the range of the average transfer errors was reduced to 12.3 to 12.7% for the DES data set.

Notable differences among the three models became evident when examining the TET-pep data set, which consist of multiple conformers representing key secondary structures observed in proteins. For this data set, the average transfer RRMSEs for RESP parameters ranged from 13.5 to 20.5%, which were notably higher than the fitting RRMSEs, which ranged from 6.7 to 10.2% with an average of 8.2%. This is consistent with the idea that fixed charge models exhibit poor transferability across different solvent environments.

In contrast, the average transfer RRMSEs of pGM-ind parameters for the TET-pep data set were in the range of 7.9 to 8.3%, approximately half of those using RESP parameters and comparable to the fitting RRMSEs, which ranged from 6.2 to 8.9%. Similarly, for pGM-perm parameters, the average transfer RRMSEs ranged from 4.2 to 4.6%, also about half of those of pGM-ind parameters, and were comparable to the fitting RRMSEs, which ranged from 3.1 to 4.3%. Given that the primary difference between RESP and pGM-ind and pGM-perm is the presence of induced dipoles in both pGM-ind and pGM-perm, we conclude that induced dipoles can significantly enhance the transferability across solvent media. Moreover, the greater improvements observed for the multiconformation TET-pep data set compared to the single-conformation DES data set suggest that for multiple conformations, pGM-ind and pGM-perm notably improves transferability over RESP. Therefore, for flexible molecules in varying dielectric environments, the inclusion of induced dipoles can be beneficial.

The substantial improvement of pGM-perm over pGM-ind, both in fitting and transferability, reinforces the idea that the permanent atomic dipoles play a critical role in accurately representing molecular ESP. This is particularly true for the multiconformation TET-pep data set, where the average RRMSEs were approximately half of those from pGM-ind. Hence, from the perspective of accuracy and transferability, we recommend including both induced and permanent multipoles in the modeling of flexible molecules and in heterogeneous solvation environments such as those pertinent to biomolecular simulations.

Among the five tested solvation environments, the gas phase ESPs consistently exhibited the largest errors when they were calculated using parameters developed in other media and the differences were quite significant. The most challenging scenario was applying the RESP parameters developed in water to calculate ESPs in gas phase, resulting in average transfer RRMSEs of 42.2 and 36.5% for DES and TET-pep data sets, respectively. These values were notably higher than the corresponding gas phase RESP fitting RRMSEs of 24.7 and 10.2%. Interestingly, all solution-phase ESPs exhibited considerably smaller errors compared with gas-phase ESPs. For example, the GAS to WAT transfer RRMSE of the DES data set was 31.6%, approximately 10.6% smaller than the transfer RRMSE from water to gas-phase (42.2%). In comparison, for solution-phase ESPs, the transfer RRMSEs using solution-phase RESP parameters ranged from 20.3 (EDC to ACT) to 26.7% (WAT to ETH), which was significantly smaller than the range of transfer RRMSEs from gas- to solution-phase RESP, which ranged from 26.0 to 31.6%. Remarkably, among the solvents, the RESP parameters derived from water ESPs consistently exhibited the poorest transferability. Therefore, given the limited transferability, it is crucial to utilize solution-phase quantum mechanics data in developing fixed charge models for solution-phase simulations, especially for highly dielectric environments such as those found in globular proteins in aqueous solution. Due to the heterogeneous dielectric environments and mobility of biomolecules, it is essential that charges be developed in the solution phase.

Both pGM-ind and pGM-perm models with induced dipoles displayed significantly improved transferability. For pGM-ind, the worst average transfer RRMSE from water to gas-phase for DES data set was 23.2%, just 1.1% greater than the gas-phase fitting RRMSE of 22.1%. For the TET-pep data set, the average transfer RRMSE from water to gas was 9.2%, only 0.3% larger than the gas-phase fitting RRMSE of 8.9%. Similarly, for pGM-perm, the average transfer RRMSE from water to gas-phase for the DES data set was 14.7%, which was 1.5% larger than the average gas-phase fitting RRMSE of 13.2%. For the TET-pep data set, the transfer RRMSE from water to gas was 4.9%, which was 0.6% larger than the gas-phase fitting error of 4.3%. Consequently, the transfer RRMSEs for both pGM-ind and pGM-perm models were consistently comparable to those for the fitting RRMSEs. This stands in contrast to the large increase in RRMSE observed from fitting to transfer with the RESP model. Clearly, the inclusion of induced dipoles significantly improves transferability across different solvents.

In addition to the significant differences among the transferability of different models, it is important to note that transferability also depends on the choice of parametrization media. For the RESP model, among the five considered media, diethyl ether (ETH) emerged as the most favorable option. In this medium, the average transfer RRMSEs were 24.3 and 13.5% for DES and TET-pep data sets, respectively. Conversely, water and gas phases proved to be the least suitable media for RESP, with average transfer RRMSEs of 28.6 and 20.5% for DES and TET-pep data sets, respectively.

In contrast, both the pGM-ind and pGM-perm models exhibited relatively consistent transferability across different media. For pGM-ind, the average transfer RRMSEs fell within narrow bands, ranging from 19.7 (gas-phase) to 20.4% (water) for the DES data set and from 7.7 (gas-phase) to 8.3% (water) for the TET-pep data set. Similarly, for pGM-perm, the average transfer RRMSEs were consistent and spanned between 12.3 (acetone) to 12.7% (water) for the DES data set and between 4.2 (acetone) and 4.6% (diethyl ether) for the TET-pep data set.

When gas-phase data were excluded to focus on solution-phase simulations, transfer RRMSEs for the RESP model ranged from 21.5 (dichloroethane) to 24.1% (water) for the DES data set and from 9.9 (dichloroethane) to 15.0% (water) for the TET-pep data set. The pGM-ind transfer RRMSEs were between 18.8 (diethyl ether) and 19.5% (water) for the DES data set and between 7.3 (diethyl ether) and 7.9% (water) for the TET-pep data set. In the case of pGM-perm, the transfer RRMSEs ranged from 11.5 (dichloroethane) and 12.0% (water) for the DES data set and from 3.9 (dichloroethane) to 4.5% (water) for the TET-pep data set. Across all of these scenarios, the gas phase and water consistently exhibited the poorest transferability, while diethyl ether and dichloroethane often emerged as the most favorable choices. It is worth noting that both the pGM-ind and pGM-perm models demonstrated relatively narrow ranges of average transfer RRMSEs, further emphasizing their consistent performance across different media.

### Transferability of Dual Solvent Models

**Figure 4:**
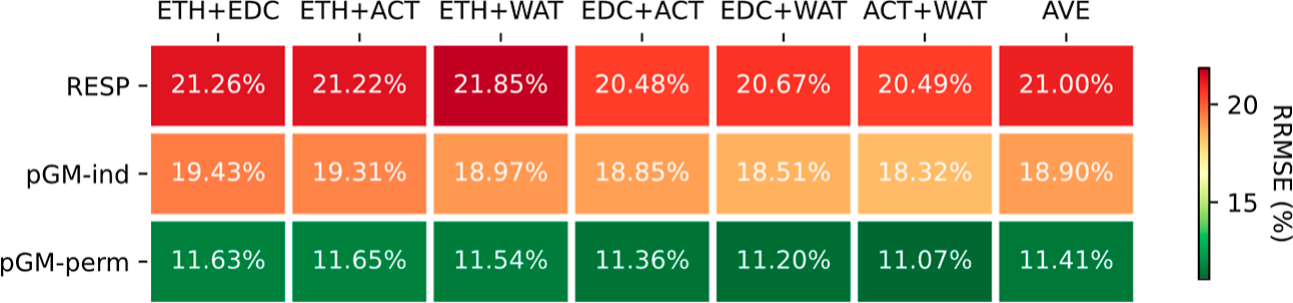
RRMSEs of the combined fitting that simultaneously considers two solvents using the DES data set.

In biomolecular simulations, macromolecules are often immersed in heterogeneous solvation environments. In addition to the highly heterogeneous dielectric environment in the lipid bilayer, the dielectric environment of the protein surface, due to proximity to water molecules, can markedly differ from the interior. To enhance model transferability, we explored the feasibility of incorporating multiple solvents into our parametrization. Figure 4 presents the statistics for combined fitting, simultaneously considering two solvents using the DES data set.

Overall, the average fitting RRMSEs were found to be comparable to those obtained from single-medium fitting. Specifically, for RESP, when gas-phase data were excluded, the average RRMSE increased to 21.0%, a mere 0.5% larger than the average for single-medium fitting (20.5%). As for pGM-ind and pGM-perm, the dual-solvent average fitting RRMSEs exhibited narrow ranges (18.3 to 19.4% for pGM-ind and 11.1 to 11.7% for pGM-perm), closely resembling the ranges observed in single-solvent average fitting RRMSEs (17.9 to 19.9% for pGM-ind and 10.8 to 11.8% for pGM-perm). Averaging across all combined fittings yielded average RRMSEs of 18.9 and 11.4%, respectively, compared to 18.9 and 11.3%, respectively, for individual medium fittings. Thus, we conclude that the combined dual-media fitting achieved quality comparable to that of the fittings conducted in individual media.

**Figure 5:**
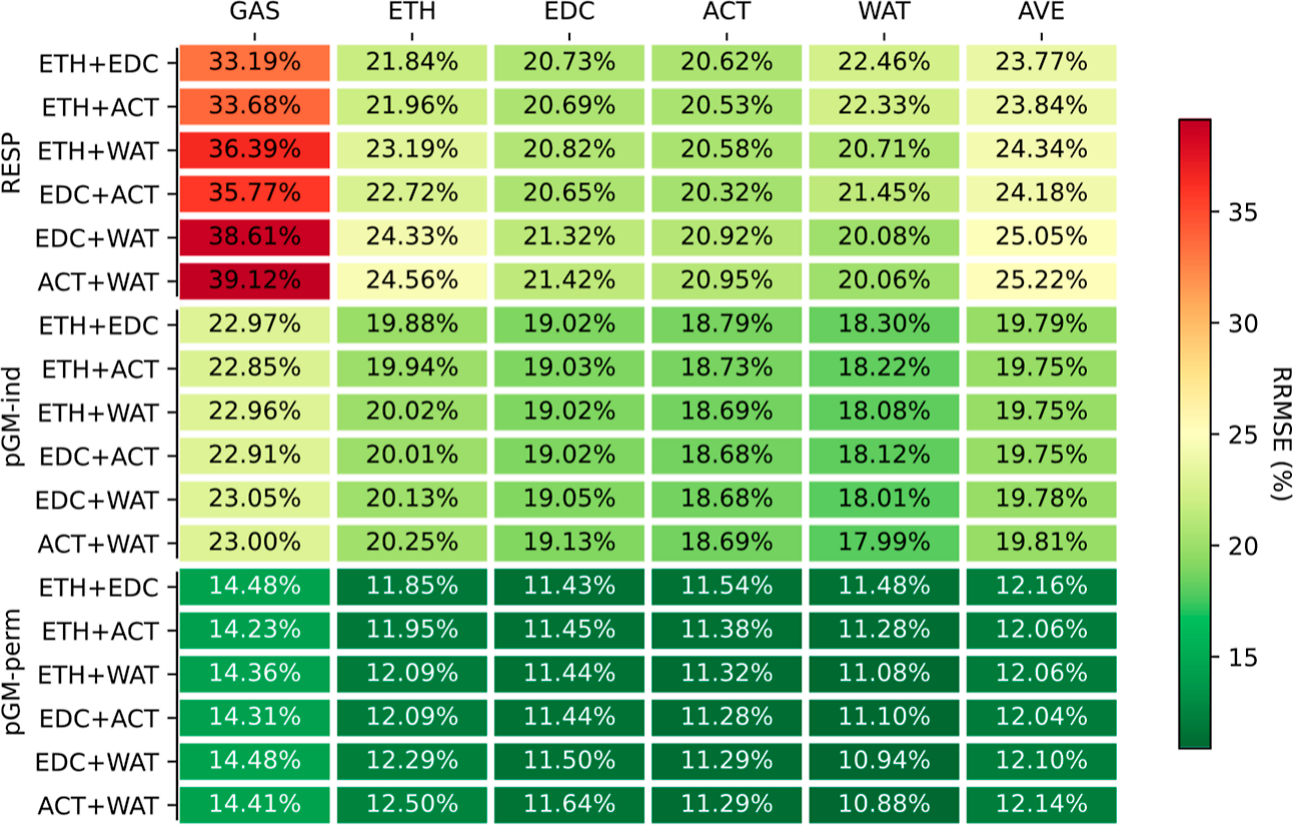
Transfer RRMSEs from six dual-solvent combined fits to single media.

To further scrutinize the parameters obtained from dual-solvent fittings, we examined their transfer performance using the ESPs calculated in five individual media, and the results are presented in Figure 5. A consistent observation is that the transfer RRMSEs were the largest when the parameters were applied to calculate the gas-phase ESPs. However, the average transfer RRMSEs showed slight improvements for all three models.

For RESP, the average transfer RRMSEs across five media were 26.3% for single-solvent parameters and 24.4% for dual solvent parameters. For pGM-ind, the average transfer RRMSEs were 20.0% for single solvent parameters and slightly improved to 19.8% for dual solvent parameters. Similarly, for pGM-perm, the average transfer RRPMSEs were 12.4% for single solvent parameters and 12.1% for dual solvent parameters. When we excluded the gas-phase ESPs, the average transfer RRMSE of the dual-solvent RESP parameters was 21.5%, compared to 22.3% for single medium parameters. The average transfer RRMSEs of dual-solvent pGM-ind and pGM-perm were 19.0 and 11.5%, respectively, slightly smaller than those of their single-solvent counterparts at 19.1 and 11.7%. Therefore, it can be concluded that dual-solvent combined fitting leads to small but consistent gains in transferability.

For pGM-perm, all six dual-solvent combined fittings achieved a similar level of average transfer RRMSEs, ranging from 11.5 to 11.6%. However, the transferability to a specific medium shows a slight dependence on the solvent media combination. For example, ACT + WAT parameters had 12.5 and 10.8% transfer RRMSEs when applied to ETH and WAT ESPs, while ETH + EDC parameters had transfer RRMSEs of 11.9 and 11.5% for ETH and WAT ESPs. Among the six dual-solvent combinations, the RRMSEs of both ETH + WAT and EDC + ACT combinations consistently came closest to the median values (12.09, 11.45, 11.31, and 11.09% for ETH, EDC, ACT, and WAT, respectively, as shown in Table S4A). Therefore, we recommend either the ETH + WAT or EDC + ACT dual-solvent approach for optimal transferability when developing the pGM-perm model.

### Comparing Results from HF/6-31G* ESP with MP2/aug-cc-pVTZ

The HF/6-31G* method has been used extensively in parametrization of fixed charge models for molecular mechanics simulations. Although the electrostatic potentials calculated using this method are formally gas-phase ESPs, the small basis set makes the dipole moments notably larger than those gas-phase dipole moments calculated with higher level methods with larger basis sets. Therefore, in practice, charges developed at the HF/6-31G* level have been widely used in condensed-phase simulations. In previous work,^11^ we assessed a wide range of quantum mechanical methods, including HF/6-31G*, and found that HF/6-31G* ESPs are poor mimics of the gas-phase ESPs calculated at either the MP2/aug-cc-pVQZ or the CCSD/aug-cc-pVQZ level. Here, we take the opportunity to compare HF/6-31G* ESPs and the resulting electrostatic parameters calculated in both gas-phase and solution-phase ESPs at the MP2/aug-cc-pVTZ level for the DES data set.

The average dipole moments obtained at the HF/6-31G* theory level fell between MP2/aug-cc-pVTZ gas-phase and MP2/aug-cc-pVTZ in diethyl ether, among the five media (Table 1). Thus, indeed, HF/6-31G* dipole moments are larger than MP2/aug-cc-pVTZ gas-phase dipole moments. In terms of RMSD, HF/6-31G* dipole moments were closest to the gas phase. Interestingly, when the parameters obtained by fitting to HF/6-31G* ESPs were applied to calculate the in-solution dipole moments and compared against those calculated at the MP2/aug-cc-pVTZ level (see Table 1), all three methods (RESP, pGM_ind, and pGM_perm) showed smaller RMSDs. For example, in diethyl ether, the RESP charge and MP2/aug-cc-pVTZ dipole moments differ by RMSD = 0.30D, down from RMSD = 0.35D between HF/6-31G* and MP2/aug-cc-pVTZ. Similarly, pGM-ind and pGM-perm differ from MP2/aug-cc-pVTZ dipole moments by 0.26D and 0.24D, respectively. The largest change was for the dipole moments in water in which the HF/6-31G* and MP2/aug-cc-pVTZ dipole moments differ by RMSD = 0.84D, which is larger than that of RESP (RMSD = 0.76D) and is more than twice as large as that of pGM-ind (RMSD = 0.36D) and pGM-perm (RMSD = 0.35D). Thus, comparison among the dipole moments suggests that RESP, pGM_ind, and pGM_perm were closer to MP2/aug-cc-pVTZ than HF/6-31G* in water.

**Table 1:** Comparison of Average Dipole Moments for DES Set Obtained at HF/6-31g* (HF) in Vacuo/Gas Phase Fitting to Those Obtained at MP2/aug-cc-pVTZ and Different Solvent Media

		HF	MP2/aug-cc-pVTZ				
		GAS	GAS	ETH	EDC	ACT	WAT
QM	AVE	2.33	2.13	2.58	2.74	2.78	3.00
	RMS	2.82	2.59	3.11	3.30	3.34	3.63
	RMSD		0.29	0.35	0.52	0.56	0.84
		FIT	Tests				
RESP	AVE	2.41	2.41	2.41	2.41	2.41	2.41
	RMS	2.91	2.91	2.91	2.91	2.91	2.91
	RMSD	0.16	0.38	0.30	0.46	0.50	0.76
pGM_ind	AVE	2.28	2.28	2.59	2.78	2.86	3.10
	RMS	2.78	2.78	3.14	3.37	3.46	3.78
	RMSD	0.17	0.29	0.26	0.28	0.31	0.36
pGM_perm	AVE	2.32	2.32	2.63	2.82	2.90	3.14
	RMS	2.81	2.81	3.18	3.41	3.50	3.81
	RMSD	0.06	0.29	0.24	0.27	0.30	0.35

Unlike dipole moments, different scenarios were observed for ESPs; see Table 2. Compared to the fittings using MP2/aug-cc-pVTZ ESPs (Figure 1), all three models achieved comparable fitting RRMSEs. For RESP, the RRMSE was 21.4% in the HF/6-31G* fitting, compared to the average 21.3% in MP2/aug-cc-pVTZ fittings. For pGM-ind and pGM-perm, the RRMSES in HF/6-31G* fittings were 21.0 and 11.2%, respectively, compared to the averages of 19.5 and 11.7%, respectively, in MP2/aug-cc-pVTZ fittings. Despite the comparable fitting RRMSEs, the transfer RRMSEs were all notably larger. In particular, the average transfer RRMSEs of pGM-perm HF/6-31G* parameters were 16.4%, notably larger than the average fitting RRMSE of 11.2%.

**Table 2:** Average RRMSEs in Reproduction of ESPs When Transferring Fitted Electrostatic Parameters From the HF/6-31g* to MP2/aug-cc-pVTZ Theory Level and Various Solvent Media

	FIT	TEST on MP2/aug-cc-pVTZ ESPs					
	HF/6-31G* (%)	GAS (%)	ETH (%)	EDC (%)	ACT (%)	WAT (%)	Ave (%)
RESP	21.41	28.89	23.89	24.49	24.75	26.93	25.79
pGM_ind	21.00	25.85	22.00	21.35	21.41	20.94	22.31
pGM_perm	11.23	19.05	15.66	15.59	15.93	15.83	16.41

Compared to the parameters obtained using MP2/aug-cc-pVTZ ESPs, the transfer RRMSEs from HF/6-31G* parameters to MP2/aug-cc-pVTZ ESPs were elevated for pGM-ind and pGM-perm models and retained at a similar level for the RESP (Figure 2). The average transfer RRMSEs of HF/6-31G* parameters were 25.8, 22.3, and 16.4%, for RESP, pGM-ind, and pGM-perm, respectively. In comparison, the average single-solvent transfer RRMSEs were 26.3, 20.0, and 12.4% for RESP, pGM-ind, and pGM-perm, respectively. Therefore, while the RESP HF/6-31G* model is as transferable as the in-media RESP MP2/aug-cc-pVTZ models, both pGM-ind and pGM-perm HF/6-31G* models are notably more inferior to the in-media counterparts.

Therefore, we conclude that while HF/6-31G* ESPs are acceptable to develop RESP fixed charge models, largely because RESP is the least transferable model among the three studied here, they are inadequate to develop transferable parameters for pGM-ind and pGM-perm models. Indeed, this observation is consistent with the conclusion we drew earlier when we compared the accuracy of ESPs from a variety of QM methods and basis sets against high-level ab initio data. Here, our conclusion serves to reinforce that idea.

## Conclusions

The transferability of force field parameters is an important attribute of a high-quality molecular mechanics force field. The accuracy and transferability of the pGM models have been evaluated to a certain extent in previous works. However, considering that most of the biomolecular simulations are performed in aqueous and heterogeneous environments, it is particularly important to investigate the quality and transferability of the models across different media. In this study, we adopted the PCM model to represent the solvent effects of five solvent media with dielectric constants ranging from 1.0 (GAS) to 78.4 (WAT).

The results obtained from testing on 377 small molecules revealed that the fitting quality of pGM-ind was slightly better than that of the RESP model and its transferability was notably better than RESP, highlighting the important role played by the induced dipoles in cross-solvent transferability. The pGM-perm, with permanent atomic dipoles, had notably better fitting quality and transferability than the RESP and pGM-ind models. Assessment conducted on a set of 20 amino acid tetrapeptides of multiple conformations showed that pGM-ind achieved consistently better fitting quality and, more importantly, reduced the average transfer RRMSE by about half from 16.4% when using RESP to 7.9%. The notably better transferability in comparison to RESP is indicative of the important roles that the induced dipoles play in both conformational and solvent transferability. Tests on the 20 amino acid tetrapeptides showed that the pGM-perm model achieved the best fitting quality and transferability among the three models, and its average transfer RRMSE (4.4%) was 56% of that of pGM-ind (7.9%) and 27% of that of RESP (16.3%).

Among the five tested media, in terms of transferability as judged by the average transfer RRMSEs, ETH and EDC are the best choices for RESP parametrization for both multiconformation tetrapeptides and single-conformation small molecules, whereas GAS and WAT are notably worse than the other three solvent media. On the other hand, the transfer performances of pGM-ind in the five media were at a similar level, and gas-phase ESPs had a slight edge. The pGM-perm model performed consistently in all five media, and ACT and EDC are slightly better than the other three.

We evaluated dual-solvent fitting strategies by combining the ESPs of the 377 small molecule DES set from two different media. All three types of models showed consistent transferability. For RESP, the average transfer RRMSEs ranged from 23.8 (ETH + EDC) to 25.2% (ACT + WAT). For pGM-ind, the average transfer RRMSEs of all six media combinations were about 19.8%. For pGM-perm, they were between 12.0 (EDC + ACT) and 12.2% (ETH + EDC). The consistent RRMSEs across multiple combinations suggest that the combined dual-solvent strategy is a robust strategy.

Comparisons were also made to the parameters developed using gas-phase HF/6-31G* ESPs of the 377 small molecule DES set. The large transfer RRMSEs ranging from 23.9 to 28.9% (RESP), 20.9 to 25.9% (pGM-ind), and 15.6 to 19.1% (pGM-perm) suggest that the gas-phase HF/6-31G* ESPs are inadequate to be used in developing the electrostatic parameters for transferable pGM-ind and pGM-perm models.

An interesting and somewhat surprising observation was the notable difference between the pGM-perm and pGM-ind models. Not only does pGM-perm demonstrate consistently and significantly better transferability than pGM-ind in all comparisons, but also the improvements of pGM-ind over RESP were consistently marginal. The observation that pGM-perm performs notably better than pGM-ind was consistent with our earlier tests of the transferability across conformations and oligomeric states. However, the marginally improved transferability of pGM-ind in comparison to RESP is difficult to explain simply by the lack of permanent dipoles which are expressed along the bonds in the CBV frame, and their presence is expected to improve conformational transferability. In this study, the conformations were intentionally kept identical throughout all of the solvent environments. Therefore, the observations can only be attributed to the distributions of the charges because only the charges are the fitting variables in both RESP and pGM-ind and there are no permanent dipoles in either pGM-ind or RESP. In other words, the static field is entirely represented by the charges. Thus, we speculate that the balance between charge and dipoles might have played roles. Furthermore, because of the absence of permanent dipoles in both pGM-ind and RESP models, one may anticipate somewhat overcompensation from the charges, leading to the scenario that resembles overfitting.

It should be noted that ESP configuration and cutoffs in both short and long ranges have been known to influence the fitting quality. Therefore, Hu et al.^25^ developed a rotationally invariant object function to minimize the effect of the abrupt cutoff and the truncation errors in ESP data. In the future, we can explore such a method.

## Supporting Information Available

Sample Gaussian input file to calculate the electrostatic potential and PCM surface charges, description of error calculations used in this study, fitting quality of three models, difference between fitting and transfer RRMSEs, and average transfer RMSEs (PDF)
