# Supplementary material for: PCMRESP: A Method for Polarizable Force Field Parameter Development and Transferability of the Polarizable Gaussian Multipole Models Across Multiple Solvents
Source: J Chem Theory Comput. 2024 Mar 19;20(7):2820–9. doi: 10.1021/acs.jctc.4c00064 (PMC11008095; doi:10.1021/acs.jctc.4c00064)
Supplement: Supplementary file 1 — ct4c00064_si_001.pdf [file ct4c00064_si_001.pdf]

Supporting Information

PCMRESP: a Method for Polarizable Force Field Parameter Development and Transferability of the Polarizable Gaussian Multipole Models Across Multiple Solvents

Yong Duan, [*,§] *Taoyu Niu*[†]*, Junmei Wang*[†]*, Piotr Cieplak,*[‡] *and Ray Luo*[▿]

[§]UC Davis Genome Center and Department of Biomedical Engineering, University of California, Davis, One Shields Avenue, Davis, California 95616, United States

[†]Department of Pharmaceutical Sciences and Computational Chemical Genomics Screening Center, School of Pharmacy, University of Pittsburgh, Pittsburgh, PA 15261, United States

[‡]SBP Medical Discovery Institute, 10901 North Torrey Pines Road, La Jolla, California 92037, United States

[▿]Departments of Molecular Biology and Biochemistry, Chemical and Biomolecular Engineering, Materials Science and Engineering, and Biomedical Engineering, University of California, Irvine. Irvine, California 92697, United States

***Corresponding Author**

Email: duan@ucdavis.edu (Yong Duan)

A sample Gaussian input to calculate electrostatic potential and PCM surface charges

```
--Link1--
% Nproc=16
% chk=CHK
% mem=58GB
# P
# maxdisk=300GB
# B3LYP/3-21G IOP(10/74=20)
# SCRF=(SMD, solvent=Water, NonEq=Save, read)
# DENSITY=current

  A test

   0    1
    O   -0.000000    0.118827    0.000000
    H    0.755858   -0.469951   -0.000000
    H   -0.755858   -0.480662   -0.000000

    GeomView

--Link1--
% Nproc=16
% chk=CHK
% mem=58GB
# P
# maxdisk=400GB
# MP2/aug-cc-pvtz
# scrf=(SMD, solvent=Water, Read, NonEQ=read, External)
# geom=allcheck nosym IOP(5/87=12) IOp(1/33=3)
# DENSITY=current

    GeomView

--Link1--
% Nproc=16
% chk=CHK
% mem=58GB
# maxdisk=400GB
# geom=allcheck nosym DENSITY=(check, current)
# Pop=(MK, ReadRadii)
# iop(6/33=2) iop(6/42=6) iop(6/43=20)

  I         1.99

  I         1.99
```

Root-Mean-Square (RMS), Root-Mean-Square Error (RMSE) and Relative Root-Mean-Square Error (RRMSE):

The RMS of a series of quantities f_i is:

$$RMS = \sqrt{\frac{1}{n}\sum_{i=1}^{n}[f_i]^2}$$

The RMSE and RRMSE of a series of quantities f_i with respect to the reference series, f_i^{ref}, are:

$$RMSE = \sqrt{\frac{1}{n}\sum_{i=1}^{n}\left[f_i - f_i^{ref}\right]^2}$$

$$RRMSE = \sqrt{\frac{\sum_{i=1}^{n}\left[f_i - f_i^{ref}\right]^2}{\sum_{i=1}^{n}\left[f_i^{ref}\right]^2}}$$

For ElectroStatic Potential (EPS), $f_i = f(\boldsymbol{x}_i)$ is the EPS at position coordinates $\boldsymbol{x}_i$ calculated by RESP, pGM-ind, or pGM-perm models, and $f_i^{ref} = f^{ref}(\boldsymbol{x}_i)$ is the EPS at the same position calculated by quantum mechanics.

Table S1. Fitting quality for three models reported as average RMSE (e/Bohr x 10^3).

		GAS	ETH	EDC	ACT	WAT	AVE
DES	RESP	2.70	2.60	2.60	2.60	2.60	2.62
	pGM-ind	2.40	2.50	2.50	2.40	2.40	2.44
	pGM-perm	1.50	1.50	1.50	1.50	1.50	1.50
TET-pep	RESP	2.90	2.70	2.60	2.60	2.50	2.66
	pGM-ind	2.40	2.60	2.50	2.40	2.30	2.44
	pGM-perm	1.20	1.20	1.20	1.20	1.20	1.20

Table S2A: Difference Between Fitting and Transfer RRMSEs of DES dataset.

		GAS	ETH	EDC	ACT	WAT	AVE
RESP	GAS		1.28%	3.27%	3.72%	6.85%	3.78%
	ETH	9.74%		-0.30%	-0.27%	2.06%	2.81%
	EDC	14.66%	1.92%		-0.26%	0.92%	4.31%
	ACT	15.96%	2.61%	0.39%		1.08%	5.01%
	WAT	22.67%	7.22%	3.48%	2.96%		9.08%
pGM-ind	GAS		-1.35%	-2.25%	-2.76%	-3.30%	-2.42%
	ETH	3.10%		-0.79%	-0.98%	-1.43%	-0.02%
	EDC	4.02%	0.94%		-0.27%	-0.81%	0.97%
	ACT	4.16%	1.44%	0.40%		-0.59%	1.35%
	WAT	5.25%	2.50%	1.30%	0.84%		2.47%
pGM-perm	GAS		0.00%	-0.48%	-0.87%	-1.02%	-0.59%
	ETH	2.68%		-0.27%	-0.09%	-0.09%	0.56%
	EDC	3.13%	0.56%		0.03%	-0.10%	0.91%
	ACT	2.96%	1.04%	0.29%		-0.23%	1.02%
	WAT	3.86%	1.97%	1.01%	0.61%		1.86%

Table S2B: Average Transfer RMSE of DES dataset (e/Bohr x 10^3).

		GAS	ETH	EDC	ACT	WAT	AVE
RESP	GAS		3.50	4.10	4.20	5.20	4.25
	ETH	3.70		2.80	2.80	3.60	3.23
	EDC	4.30	2.80		2.60	3.10	3.20
	ACT	4.40	2.90	2.60		3.10	3.25
	WAT	5.40	3.70	3.10	3.10		3.83
pGM-ind	GAS		2.60	2.60	2.60	2.60	2.60
	ETH	2.50		2.50	2.50	2.50	2.50
	EDC	2.50	2.50		2.50	2.50	2.50
	ACT	2.50	2.50	2.50		2.50	2.50
	WAT	2.50	2.60	2.50	2.50		2.53
pGM-perm	GAS		1.80	1.80	1.70	1.80	1.78
	ETH	1.70		1.60	1.60	1.70	1.65
	EDC	1.70	1.60		1.60	1.60	1.63
	ACT	1.60	1.60	1.60		1.60	1.60
	WAT	1.70	1.70	1.60	1.60		1.65

Table S2C: Difference Between Fitting and Transfer RMSEs of DES dataset (e/Bohr x 10^3).

		GAS	ETH	EDC	ACT	WAT	AVE
RESP	GAS		0.80	1.40	1.50	2.50	1.55
	ETH	1.10		0.20	0.20	1.00	0.63
	EDC	1.70	0.20		0.00	0.50	0.60
	ACT	1.80	0.30	0.00		0.50	0.65
	WAT	2.80	1.10	0.50	0.50		1.23
pGM-ind	GAS		0.20	0.20	0.20	0.20	0.20
	ETH	0.00		0.00	0.00	0.00	0.00
	EDC	0.00	0.00		0.00	0.00	0.00
	ACT	0.10	0.10	0.10		0.10	0.10
	WAT	0.10	0.20	0.10	0.10		0.13
pGM-perm	GAS		0.30	0.30	0.20	0.30	0.28
	ETH	0.20		0.10	0.10	0.20	0.15
	EDC	0.20	0.10		0.10	0.10	0.13
	ACT	0.10	0.10	0.10		0.10	0.10
	WAT	0.20	0.20	0.10	0.10		0.15

Table S3A: Difference Between Fitting and Transfer RRMSEs of TET-pep dataset.

		GAS	ETH	EDC	ACT	WAT	AVE
RESP	GAS		5.63%	9.35%	9.98%	16.20%	10.29%
	ETH	10.22%		1.00%	1.43%	7.49%	5.04%
	EDC	16.61%	2.39%		0.00%	4.07%	5.77%
	ACT	17.68%	3.02%	0.16%		3.71%	6.14%
	WAT	29.81%	12.26%	6.61%	6.00%		13.67%
pGM-ind	GAS		0.04%	-0.94%	-1.56%	-2.50%	-1.24%
	ETH	1.81%		-0.65%	-0.62%	-1.26%	-0.18%
	EDC	2.22%	0.78%		-0.13%	-0.85%	0.51%
	ACT	2.05%	1.35%	0.41%		-0.85%	0.74%
	WAT	2.97%	2.60%	1.55%	1.00%		2.03%
pGM-perm	GAS		1.23%	0.46%	-0.26%	-0.67%	0.19%
	ETH	2.16%		-0.15%	0.31%	0.41%	0.68%
	EDC	1.92%	0.44%		0.16%	0.15%	0.67%
	ACT	1.25%	1.16%	0.41%		-0.25%	0.64%
	WAT	1.72%	2.21%	1.27%	0.58%		1.45%

Table S3B: Average Transfer RMSE of TET-pep dataset (e/Bohr x 10^3).

		GAS	ETH	EDC	ACT	WAT	AVE
RESP	GAS		5.10	6.60	6.90	9.80	7.10
	ETH	5.20		3.20	3.30	5.90	4.40
	EDC	6.70	3.20		2.60	4.40	4.23
	ACT	7.00	3.40	2.60		4.20	4.30
	WAT	9.90	6.00	4.40	4.30		6.15
pGM-ind	GAS		2.80	2.70	2.50	2.40	2.60
	ETH	2.70		2.50	2.50	2.60	2.58
	EDC	2.60	2.60		2.50	2.50	2.55
	ACT	2.50	2.70	2.50		2.40	2.53
	WAT	2.50	2.80	2.60	2.40		2.58
pGM-perm	GAS		1.80	1.60	1.40	1.40	1.55
	ETH	1.70		1.30	1.40	1.60	1.50
	EDC	1.50	1.30		1.30	1.40	1.38
	ACT	1.30	1.50	1.30		1.20	1.33
	WAT	1.30	1.70	1.50	1.30		1.45

Table S3C: Difference Between Fitting and Transfer RMSEs of TET-pep dataset (e/Bohr x 10^3).

		GAS	ETH	EDC	ACT	WAT	AVE
RESP	GAS		2.20	3.70	4.00	6.90	4.20
	ETH	2.50		0.50	0.60	3.20	1.70
	EDC	4.10	0.60		0.00	1.80	1.63
	ACT	4.40	0.80	0.00		1.60	1.70
	WAT	7.40	3.50	1.90	1.80		3.65
pGM-ind	GAS		0.40	0.30	0.10	0.00	0.20
	ETH	0.10		-0.10	-0.10	0.00	-0.03
	EDC	0.10	0.10		0.00	0.00	0.05
	ACT	0.10	0.30	0.10		0.00	0.13
	WAT	0.20	0.50	0.30	0.10		0.28
pGM-perm	GAS		0.60	0.40	0.20	0.20	0.35
	ETH	0.50		0.10	0.20	0.40	0.30
	EDC	0.30	0.10		0.10	0.20	0.18
	ACT	0.10	0.30	0.10		0.00	0.13
	WAT	0.10	0.50	0.30	0.10		0.25

Table S4. Average fitting RMSEs for three models in dual solvents (e/Bohr x 10^3).

	ETH+EDC	ETH+ACT	ETH+WAT	EDC+ACT	EDC+WAT	ACT+WAT	AVE.
RESP	2.70	2.70	2.90	2.60	2.80	2.80	2.75
pGM-ind	2.50	2.50	2.50	2.50	2.50	2.40	2.48
pGM-perm	1.50	1.60	1.60	1.60	1.60	1.60	1.58

Table S5A: Average Transfer RRMSE of DES dataset from dual-solvent parameters.

		GAS	ETH	EDC	ACT	WAT	AVE.
RESP	ETH+EDC	33.19%	21.84%	20.73%	20.62%	22.46%	23.77%
	ETH+ACT	33.68%	21.96%	20.69%	20.53%	22.33%	23.84%
	ETH+WAT	36.39%	23.19%	20.82%	20.58%	20.71%	24.34%
	EDC+ACT	35.77%	22.72%	20.65%	20.32%	21.45%	24.18%
	EDC+WAT	38.61%	24.33%	21.32%	20.92%	20.08%	25.05%
	ACT+WAT	39.12%	24.56%	21.42%	20.95%	20.06%	25.22%
	Median	36.08%	22.96%	20.78%	20.60%	21.08%	
pGM-ind	ETH+EDC	22.97%	19.88%	19.02%	18.79%	18.30%	19.79%
	ETH+ACT	22.85%	19.94%	19.03%	18.73%	18.22%	19.75%
	ETH+WAT	22.96%	20.02%	19.02%	18.69%	18.08%	19.75%
	EDC+ACT	22.91%	20.01%	19.02%	18.68%	18.12%	19.75%
	EDC+WAT	23.05%	20.13%	19.05%	18.68%	18.01%	19.78%
	ACT+WAT	23.00%	20.25%	19.13%	18.69%	17.99%	19.81%
	Median	22.97%	20.02%	19.03%	18.69%	18.10%	
pGM-perm	ETH+EDC	14.48%	11.85%	11.43%	11.54%	11.48%	12.16%
	ETH+ACT	14.23%	11.95%	11.45%	11.38%	11.28%	12.06%
	ETH+WAT	14.36%	12.09%	11.44%	11.32%	11.08%	12.06%
	EDC+ACT	14.31%	12.09%	11.44%	11.28%	11.10%	12.04%
	EDC+WAT	14.48%	12.29%	11.50%	11.29%	10.94%	12.10%
	ACT+WAT	14.41%	12.50%	11.64%	11.29%	10.88%	12.14%
	Median	14.39%	12.09%	11.45%	11.31%	11.09%	

Table S5B: Difference between fitting and transfer RRMSEs of DES dataset from dual-solvent parameters.

		GAS	ETH	EDC	ACT	WAT	AVE.
RESP	ETH+EDC	11.93%	0.58%	-0.53%	-0.64%	1.20%	2.51%
	ETH+ACT	12.46%	0.74%	-0.53%	-0.69%	1.11%	2.62%
	ETH+WAT	14.54%	1.34%	-1.03%	-1.27%	-1.14%	2.49%
	EDC+ACT	15.29%	2.24%	0.17%	-0.16%	0.97%	3.70%
	EDC+WAT	17.94%	3.66%	0.65%	0.25%	-0.59%	4.38%
	ACT+WAT	18.63%	4.07%	0.93%	0.46%	-0.43%	4.73%
pGM-ind	ETH+EDC	3.54%	0.45%	-0.41%	-0.64%	-1.13%	0.36%
	ETH+ACT	3.54%	0.63%	-0.28%	-0.58%	-1.09%	0.44%
	ETH+WAT	3.99%	1.05%	0.05%	-0.28%	-0.89%	0.78%
	EDC+ACT	4.06%	1.16%	0.17%	-0.17%	-0.73%	0.90%
	EDC+WAT	4.54%	1.62%	0.54%	0.17%	-0.50%	1.27%
	ACT+WAT	4.68%	1.93%	0.81%	0.37%	-0.33%	1.49%
pGM-perm	ETH+EDC	2.85%	0.22%	-0.20%	-0.09%	-0.15%	0.53%
	ETH+ACT	2.58%	0.30%	-0.20%	-0.27%	-0.37%	0.41%
	ETH+WAT	2.82%	0.55%	-0.10%	-0.22%	-0.46%	0.52%
	EDC+ACT	2.95%	0.73%	0.08%	-0.08%	-0.26%	0.68%
	EDC+WAT	3.28%	1.09%	0.30%	0.09%	-0.26%	0.90%
	ACT+WAT	3.34%	1.43%	0.57%	0.22%	-0.19%	1.07%

Table S5C: Transfer RMSEs of DES dataset from dual-solvent parameters (e/Bohr x 10^3).

		GAS	ETH	EDC	ACT	WAT	AVE.
RESP	ETH+EDC	4.00	2.70	2.70	2.70	3.30	3.08
	ETH+ACT	4.00	2.70	2.60	2.70	3.30	3.06
	ETH+WAT	4.50	3.00	2.70	2.70	2.90	3.16
	EDC+ACT	4.30	2.80	2.60	2.60	3.10	3.08
	EDC+WAT	4.80	3.20	2.80	2.80	2.80	3.28
	ACT+WAT	4.90	3.20	2.80	2.80	2.80	3.30
pGM-ind	ETH+EDC	2.50	2.50	2.50	2.50	2.50	2.50
	ETH+ACT	2.50	2.50	2.50	2.50	2.50	2.50
	ETH+WAT	2.50	2.50	2.50	2.40	2.50	2.48
	EDC+ACT	2.50	2.50	2.50	2.40	2.50	2.48
	EDC+WAT	2.50	2.50	2.50	2.40	2.40	2.46
	ACT+WAT	2.50	2.50	2.50	2.40	2.40	2.46
pGM-perm	ETH+EDC	1.70	1.50	1.60	1.60	1.70	1.62
	ETH+ACT	1.60	1.60	1.60	1.60	1.60	1.60
	ETH+WAT	1.60	1.60	1.60	1.60	1.60	1.60
	EDC+ACT	1.60	1.60	1.60	1.50	1.60	1.58
	EDC+WAT	1.70	1.60	1.60	1.50	1.60	1.60
	ACT+WAT	1.60	1.70	1.60	1.60	1.60	1.62

Table S5D: Differences between fit and transfer RMSEs of DES dataset from dual-solvent parameters (e/Bohr x 10^3).

		GAS	ETH	EDC	ACT	WAT	AVE.
RESP	ETH+EDC	1.30	0.00	0.00	0.00	0.60	0.38
	ETH+ACT	1.30	0.00	-0.10	0.00	0.60	0.36
	ETH+WAT	1.60	0.10	-0.20	-0.20	0.00	0.26
	EDC+ACT	1.70	0.20	0.00	0.00	0.50	0.48
	EDC+WAT	2.00	0.40	0.00	0.00	0.00	0.48
	ACT+WAT	2.10	0.40	0.00	0.00	0.00	0.50
pGM-ind	ETH+EDC	0.00	0.00	0.00	0.00	0.00	0.00
	ETH+ACT	0.00	0.00	0.00	0.00	0.00	0.00
	ETH+WAT	0.00	0.00	0.00	-0.10	0.00	-0.02
	EDC+ACT	0.00	0.00	0.00	-0.10	0.00	-0.02
	EDC+WAT	0.00	0.00	0.00	-0.10	-0.10	-0.04
	ACT+WAT	0.10	0.10	0.10	0.00	0.00	0.06
pGM-perm	ETH+EDC	0.20	0.00	0.10	0.10	0.20	0.12
	ETH+ACT	0.00	0.00	0.00	0.00	0.00	0.00
	ETH+WAT	0.00	0.00	0.00	0.00	0.00	0.00
	EDC+ACT	0.00	0.00	0.00	-0.10	0.00	-0.02
	EDC+WAT	0.10	0.00	0.00	-0.10	0.00	0.00
	ACT+WAT	0.00	0.10	0.00	0.00	0.00	0.02
